# Beyond the hospital walls: community-based approaches to leukemia management in older adults

**DOI:** 10.1097/MS9.0000000000004728

**Published:** 2026-01-20

**Authors:** Emmanuel Ifeanyi Obeagu, Aakib Rahman Parray

**Affiliations:** a Department of Biomedical and Laboratory Science, Africa University, Mutare, Zimbabwe; b Department of Molecular Medicine and Haematology, Faculty of Health Sciences, University of the Witwatersrand, Johannesburg, South Africa; c Department of Psychology, Akal University, Talwandi Sabo, India

**Keywords:** aging population, community-based care, leukemia management, older adults, supportive care

## Abstract

Leukemia incidence among older adults is rising globally, presenting complex challenges that extend beyond conventional hospital-based treatment. Older patients often face multiple comorbidities, functional impairments, and social barriers that complicate disease management and diminish quality of life. This review explores the critical role of community-based care models that provide decentralized, patient-centered leukemia management tailored to the unique needs of the aging population. Community-based approaches encompass outpatient services, home healthcare, telemedicine, and psychosocial support programs designed to improve accessibility, continuity, and holistic care. These models enable early detection of complications, personalized symptom management, and integration of rehabilitation services, thereby reducing hospitalizations and enhancing patient independence. Moreover, leveraging digital health technologies further facilitates real-time monitoring and remote consultations, addressing logistical challenges for frail older adults. Public health policies supporting community-based leukemia care are essential for sustainable healthcare delivery amid demographic shifts. Investment in workforce training, infrastructure, and equitable access to services can optimize outcomes for elderly leukemia patients while alleviating pressure on hospital resources. As healthcare systems evolve, embracing community-centered strategies will be pivotal in improving survival, quality of life, and overall health equity for older adults living with leukemia.

## Introduction

Leukemia remains a significant public health concern, particularly among older adults, who represent the majority of new diagnoses worldwide[1]. The aging immune system, accumulation of genetic mutations, and exposure to environmental factors contribute to the higher incidence and unique disease biology in this population^[2,3]^. Older patients with leukemia often contend with multiple comorbidities, decreased physiological reserves, and increased vulnerability to treatment toxicities, which complicate traditional hospital-based management strategies^[4,5]^. As the global population ages, healthcare systems must adapt to these evolving challenges by exploring innovative care models that extend beyond the confines of hospitals. Hospital-centered leukemia care, while essential for diagnosis and intensive treatments[6], can impose substantial burdens on elderly patients[7]. Frequent hospital visits, prolonged admissions, and complex chemotherapy regimens may exacerbate frailty, contribute to nosocomial infections, and impair psychosocial well-being[8]. Additionally, transportation difficulties and social isolation often limit access to hospital services for many older adults, particularly those living in rural or underserved areas^[9,10]^. These factors underscore the necessity of alternative management approaches that provide comprehensive, accessible, and patient-centered care in community settings[11].

**HIGHLIGHTS**

- Home-based chemotherapy improves adherence, reduces hospital visits, and enhances quality of life for older adults with leukemia.
- Telehealth ensures timely specialist access and real-time symptom monitoring.
- Community nursing provides personalized care and psychosocial support.
- Hospice integration complements hospital-based treatment, optimizing symptom management.
- Community-based models are feasible globally, including resource-limited and aging populations.

Community-based care models offer a promising avenue for addressing the multidimensional needs of older leukemia patients[12]. By decentralizing services to outpatient clinics, home health care, and telemedicine platforms, these approaches facilitate early detection of complications, continuous symptom monitoring, and timely intervention without the need for frequent hospital stays^[13,14]^. Moreover, community programs can integrate psychosocial support, rehabilitation, and caregiver education, which are critical components in managing the holistic impact of leukemia on older adults’ lives[15]. The integration of technology within community-based care has further expanded the potential to improve leukemia management[16]. Tele-health platforms enable virtual consultations, remote monitoring of vital signs and laboratory values, and digital symptom tracking, reducing the physical and emotional strain associated with hospital visits[17]. These tools are especially valuable for frail elderly patients who may face mobility challenges or live far from specialized treatment centers[18]. Consequently, technology-enhanced community care can promote treatment adherence, timely adjustments, and better overall outcomes[19].

**Figure 1.**
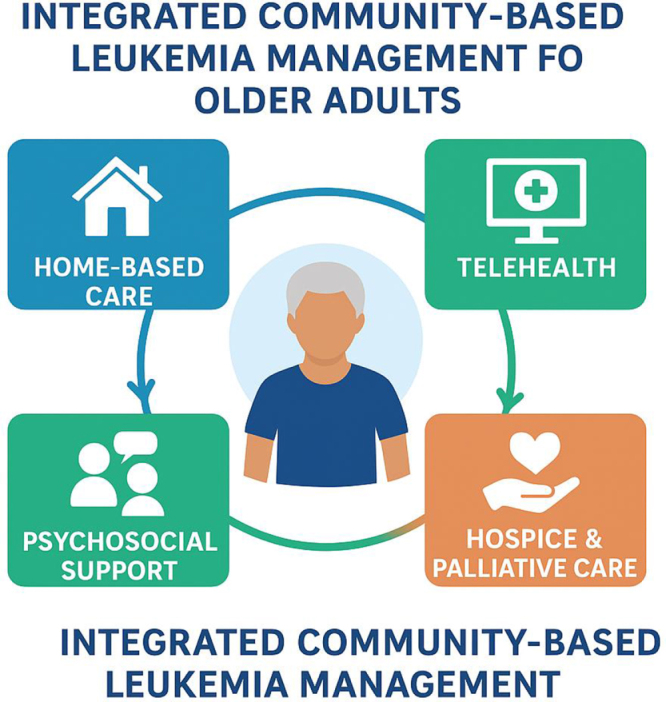
The integrated community-based leukemia management framework for older adults.

Beyond individual patient benefits, community-based leukemia care aligns with broader public health goals of sustainability and equity[20]. By reducing hospital admissions and emergency visits, such models can alleviate pressure on healthcare infrastructure and reduce costs^[21,22]^. Additionally, they promote health equity by improving access for marginalized populations, including those in rural or low-resource settings[23]. Public health policies that support the expansion and integration of community services are therefore vital to address the rising leukemia burden in aging societies[17]. However, implementing effective community-based leukemia management faces challenges, including workforce shortages, variable technology access, and the need for standardized care protocols. Collaboration among healthcare providers, policymakers, and community organizations is essential to overcome these barriers[23]. Training programs must equip healthcare workers with geriatric and oncology expertise, while health systems need to invest in infrastructure that supports seamless care coordination and quality assurance outside hospital environments[24]. This review aims to synthesize current evidence and best practices regarding community-based approaches to leukemia management in older adults (Fig. 1).

## Aim

This review aims to explore and critically evaluate community-based approaches to leukemia management in older adults. It seeks to highlight strategies that extend care beyond hospital settings, focusing on improving accessibility, continuity, and quality of life for elderly patients.

## Methods

A comprehensive narrative review was conducted to synthesize current evidence on community-based approaches to leukemia management in older adults. Literature published between *2000 and 2025* was retrieved from *PubMed, Scopus, and Google Scholar*. Search terms included: *“community-based care,” “home chemotherapy,” “telehealth,” “older adults,” “leukemia,”* and *“community nursing.”* Boolean operators (AND, OR) were applied to refine the search.

*Inclusion criteria* were:

1. Studies involving adults aged ≥60 years diagnosed with leukemia.
2. Research reporting community-based interventions, including home chemotherapy, telehealth, nurse-led programs, or patient self-management initiatives.
3. Original research articles, systematic reviews, or observational studies published in English.

*Exclusion criteria* were:

1. Studies focusing solely on pediatric or adolescent populations.
2. Reports on non-leukemic hematologic disorders.
3. Case reports with insufficient data on community-based care.

The review followed a *structured narrative synthesis approach*, grouping findings into thematic domains:

1. Home-based chemotherapy administration
2. Telehealth and remote monitoring
3. Community nursing programs
4. Patient self-management and education

Data were extracted and analyzed for clinical relevance, feasibility, safety, and impact on quality of life, treatment adherence, and healthcare utilization. Wherever available, outcome measures were compared across studies to identify trends and evidence gaps. This approach allowed for a comprehensive, clinically focused overview of *community-centered leukemia care*, emphasizing practical implications for older adults.

### Community-based care models in leukemia management

Community-based care models represent a transformative approach to leukemia management, particularly for older adults who face unique challenges related to aging and disease complexity[25]. These models prioritize delivering care outside traditional hospital settings, focusing on outpatient services, home-based care, and the use of telemedicine to enhance accessibility and patient convenience^[12,26]^. By decentralizing leukemia treatment and monitoring, community-based approaches reduce the need for frequent hospital visits, lowering the risk of hospital-acquired infections and minimizing disruptions to patients’ daily lives^[27,28]^. One prominent example of community-based care is home healthcare, where trained oncology nurses administer chemotherapy, manage transfusions, and provide symptom control directly in patients’ homes^[29,30]^. This personalized care approach supports early identification of complications, timely intervention, and adherence to treatment protocols while allowing patients to remain in a familiar, comfortable environment^[31,32]^. Additionally, home care reduces caregiver burden and transportation barriers, which are significant concerns for elderly patients with limited mobility^[33,34]^. Outpatient clinics and infusion centers embedded within community settings also play a crucial role in delivering leukemia care[35]. These facilities offer regular monitoring, laboratory testing, and administration of treatments with shorter wait times and greater convenience than hospital outpatient departments[36]. The integration of tele-health services further enhances community-based care by enabling virtual consultations, remote symptom tracking, and real-time communication between patients and healthcare providers[37]. This technology-driven approach facilitates continuous care coordination and rapid response to clinical changes, contributing to improved treatment outcomes and patient satisfaction[38].

### Psychosocial support and rehabilitation

The psychosocial impact of leukemia on older adults is profound and multifaceted, often exacerbating the physical challenges posed by the disease and its treatment^[39,40]^. Many elderly patients experience anxiety, depression, social isolation, and cognitive decline, all of which can negatively influence treatment adherence, symptom management, and overall quality of life^[41,42]^. Community-based care models recognize the critical importance of integrating psychosocial support services to address these emotional and social needs alongside medical treatment[12]. Support groups, counseling services, and caregiver education programs form the backbone of psychosocial interventions within the community^[15,43]^. Peer support groups provide a platform for patients and caregivers to share experiences, reduce feelings of isolation, and foster emotional resilience^[44,45]^. Mental health professionals working within community settings can offer tailored counseling that addresses age-specific concerns, including coping with loss of independence, fear of mortality, and changes in family dynamics^[46,47]^. Importantly, educating caregivers on symptom recognition, medication management, and emotional support strengthens the overall care network surrounding the patient[15].

Rehabilitation services are equally vital for maintaining and improving functional status in older leukemia patients^[48,49]^. Treatment-related fatigue, muscle weakness, and cognitive impairments contribute to reduced physical activity and increased risk of falls, frailty, and disability^[50,51]^. Community-based physical and occupational therapy programs help patients regain strength, enhance mobility, and adapt to daily living activities[52]. These interventions not only improve physical function but also promote psychological well-being and social engagement^[50,53]^. Tailoring rehabilitation to individual needs and integrating it with medical and psychosocial care ensures a holistic approach that supports aging adults throughout their leukemia journey.

### Public health implications and policy considerations

The increasing prevalence of leukemia among older adults poses significant public health challenges that extend beyond clinical management. Community-based care models offer promising solutions by improving healthcare accessibility, reducing hospital burden, and enhancing patient-centered outcomes[17]. However, scaling these models requires strategic policy interventions and resource allocation to ensure equitable service delivery across diverse populations, including those in rural and underserved regions. Public health systems must prioritize the integration of community-based leukemia care into broader aging and chronic disease management frameworks[12]. This includes investing in workforce development to train healthcare professionals in geriatric oncology and community health competencies[54]. Equipping home care providers, social workers, and rehabilitation specialists with specialized skills is essential for delivering comprehensive, age-appropriate leukemia care outside hospital settings^[50,55,56]^. Additionally, policies that promote intersectoral collaboration between healthcare providers, social services, and community organizations are critical to creating coordinated care networks that address the complex needs of elderly patients[57].

Technology infrastructure also demands policy attention. Expanding access to telehealth and digital health tools can mitigate barriers related to distance, mobility, and healthcare capacity[58]. Ensuring affordability, connectivity, and digital literacy among older adults are necessary steps to maximize the benefits of technology-enhanced community care[59]. Furthermore, health systems should establish standardized protocols and quality assurance mechanisms to monitor the effectiveness and safety of community-based leukemia management[17]. Policy frameworks must incorporate patient and caregiver voices to align services with real-world needs and preferences^[59,60]^. Supporting caregiver training, respite care, and psychosocial resources can improve care sustainability and patient quality of life^[60,61]^. By adopting comprehensive, patient-centered policies that facilitate community-based leukemia care, public health systems can better respond to the growing demands of aging populations and improve health equity and outcomes for older adults living with leukemia^[61,62]^.

### Community-based leukemia management in resource-limited settings: the African context

Leukemia management in resource-limited settings, particularly across Africa, presents unique challenges that require context-specific strategies. Limited access to healthcare facilities, a shortage of trained hematology and oncology personnel, and financial constraints significantly hinder timely diagnosis, treatment, and follow-up care for older adults. Infrastructure limitations, including inadequate laboratory services and insufficient supply of chemotherapy drugs, further exacerbate disparities in outcomes[63].

Community-based approaches can mitigate some of these barriers by leveraging local resources. Home-based care programs, telehealth initiatives, and community health workers can provide essential monitoring, treatment adherence support, and psychosocial care, reducing the need for frequent hospital visits. However, successful implementation requires careful adaptation to local contexts, including consideration of cultural factors, literacy levels, and regional disease burden. Partnerships with local healthcare providers, non-governmental organizations, and governmental health systems are critical to ensuring sustainability and equitable access (Table 1)[64].

**Table 1:** Community-based leukemia programs across different countries

Country/region	Intervention	Target population	Key outcomes	Lessons learned
United Kingdom	Home-based chemotherapy	Older adults with leukemia	Reduced hospital admissions, improved patient satisfaction, maintained treatment adherence	Rigorous safety protocols and nurse training are essential; effective communication with hospital teams is critical
Australia	Tele-oncology consultations	Rural and older leukemia patients	Increased access to specialist care, timely management of side effects, improved adherence	Telehealth reduces travel burden; requires reliable internet and technology support
Canada	Community nursing + psychosocial support	Older adults in urban and rural areas	Reduced emergency visits, improved quality of life, better symptom management	Multidisciplinary coordination enhances effectiveness; caregiver involvement improves outcomes
United States	Integrated home care + telehealth	Older leukemia patients with comorbidities	Improved treatment adherence, early complication detection, enhanced patient engagement	Integration of telehealth and home visits allows for personalized care; cost-effectiveness needs further evaluation
Kenya	Community health worker support + basic chemotherapy follow-up	Older adults in resource-limited settings	Improved adherence, better patient education, reduced delays in reporting symptoms	Training local health workers critical; program success depends on government and NGO support
India	Home visits + psychosocial counseling	Older adults with leukemia in urban slums	Improved quality of life, reduced hospital readmissions	Cultural adaptation and caregiver education key to success; local resource utilization improves feasibility
Sweden	Telehealth + community nursing	Older leukemia patients	High patient satisfaction, maintained treatment continuity, reduced hospital dependency	Early integration of telehealth and regular nurse visits enhances outcomes; patient digital literacy important
South Africa	Community palliative care + home-based support	Older adults with advanced leukemia	Improved symptom control, better quality of life, reduced hospital stays	Collaboration with NGOs and hospice organizations strengthens community reach; resource limitations remain a barrier

### Hospice and palliative care in community-based leukemia management

Hospice and palliative care play a crucial complementary role in the management of older adults with leukemia, particularly when integrated into community-based care models. These services focus on symptom control, psychosocial support, and quality-of-life optimization, addressing needs that often extend beyond the capabilities of hospital-based interventions[65]. In community settings, palliative care teams – including nurses, social workers, and trained volunteers – can provide home visits, pain and symptom management, medication monitoring, and caregiver education. This approach reduces unnecessary hospitalizations and emergency visits, allowing patients to receive care in familiar, supportive environments. Hospice programs further support end-of-life care planning, advance directives, and emotional support for patients and their families, ensuring a dignified and patient-centered approach[66].

By integrating hospice and palliative care with hospital-based treatment plans, community-based leukemia management can achieve a holistic balance – combining disease-directed therapy with supportive care tailored to the patient’s functional status, preferences, and social context. This model is particularly valuable for older adults who may have limited tolerance for intensive therapies but still benefit from coordinated and compassionate care[67].

### Home-based chemotherapy

Home-based chemotherapy is an emerging strategy to deliver cancer treatment safely outside hospital settings, particularly beneficial for older adults with leukemia who face mobility, transportation, or frailty challenges. Pilot initiatives globally have demonstrated the feasibility and safety of administering select chemotherapeutic regimens at home, accompanied by trained oncology nurses and strict adherence to safety protocols. Safety protocols in home-based chemotherapy typically include pre-treatment assessment of vital signs and laboratory parameters, secure drug handling and storage, management of infusion equipment, and immediate reporting mechanisms for adverse events. Infection control measures and clear communication pathways with hospital-based oncologists are essential to ensure patient safety. Pilot initiatives in countries such as the United Kingdom, Australia, and Canada have shown reductions in hospital admissions, improved patient satisfaction, and enhanced quality of life. For older adults with leukemia, home-based chemotherapy programs can maintain continuity of care, reduce the burden of travel, and support adherence to treatment regimens while allowing patients to remain in familiar, supportive environments^[68,69]^.

### Telehealth interventions

Telehealth has transformed cancer care delivery by enabling remote consultations, monitoring, and care coordination, making it particularly valuable for older adults with leukemia in both urban and rural settings. Evidence from global tele-oncology programs indicates that telehealth interventions can effectively complement hospital-based care, improving access to specialist consultations, real-time symptom monitoring, and medication adherence support. Programs in the United States, Europe, and Asia have shown that telemedicine reduces missed appointments, enables timely management of adverse effects, and enhances patient engagement. Mobile health applications, video consultations, and remote laboratory reporting systems facilitate continuous communication between patients, caregivers, and healthcare teams. Outcomes of telehealth interventions include improved adherence to treatment protocols, early detection of complications, reduced hospitalizations, and increased patient satisfaction. Additionally, telehealth provides an opportunity to integrate multidisciplinary support – including dietitians, social workers, and palliative care specialists – into a single coordinated platform, improving overall leukemia management for older adults^[70,71]^.

### Psychosocial support and community nursing programs

Psychosocial support and community nursing programs are integral components of community-based leukemia management, addressing the emotional, cognitive, and social needs of older adults undergoing treatment. Community nurses serve as primary points of contact, conducting home visits, monitoring laboratory results, administering medications, and coordinating care across multiple providers. Structured psychosocial interventions include counseling, caregiver education, peer support groups, and practical assistance with transportation, nutrition, and medication adherence. Evidence shows that such interventions significantly improve treatment adherence, reduce anxiety and depression, and enhance patient-reported quality of life. Pilot studies in diverse settings demonstrate that community-based nursing and psychosocial support can reduce emergency visits, prevent treatment interruptions, and increase patient engagement in decision-making. By providing tailored, patient-centered support, these programs bridge gaps between hospital care and community resources, enabling older adults with leukemia to receive comprehensive care in their homes and communities^[72,73]^.

### Challenges

Despite the promise of community-based approaches to leukemia management in older adults, several challenges hinder their widespread implementation and effectiveness. One of the primary barriers is the limited availability of trained healthcare professionals with expertise in both oncology and geriatrics. Community health settings often lack access to hematologists, specialized nurses, and geriatricians, making it difficult to deliver comprehensive leukemia care outside of hospital environments^[63,64]^. This shortage is especially acute in low-resource or rural areas, where workforce constraints are most pronounced. Another major challenge lies in the fragmentation of care across different providers and settings. Without clear communication channels and coordinated treatment plans, patients may experience gaps in care continuity, leading to delayed interventions, medication errors, and unnecessary hospital readmissions[65]. Older adults, who often face cognitive impairments and mobility issues, are particularly vulnerable to such discontinuities. Moreover, the absence of standardized protocols for community-based leukemia management makes it difficult to monitor care quality and ensure consistent treatment outcomes across diverse health systems^[16,66]^.

Logistical and financial barriers also impede access to community-based services. Many elderly patients encounter difficulties related to transportation, home modification needs, or out-of-pocket costs for services not covered by insurance.^[67–69]^ Additionally, not all communities have the infrastructure or digital capacity to support telemedicine, remote monitoring, or home infusion therapies[70]. These inequities can result in disparities in leukemia outcomes among socioeconomically disadvantaged and geographically isolated populations. Cultural factors, stigma, and lack of awareness about leukemia and aging can further complicate community engagement^[71,74]^. Patients and caregivers may be hesitant to accept non-hospital care, fearing it is less effective or lacking in oversight[72]. Building trust in community-based systems, therefore, requires sustained efforts in patient education, cultural sensitivity, and community outreach^[73,75]^. Addressing these challenges necessitates systemic change – strengthening primary care networks, expanding training programs, ensuring equitable resource distribution, and fostering collaboration across all levels of care[17]. Without tackling these critical barriers, the full potential of community-based leukemia management for older adults will remain unrealized.

## Future directions

While community-based approaches to leukemia management in older adults show promising results, there remains a critical need for rigorous research to establish their efficacy, feasibility, and sustainability. Future studies should focus on:

1. *Randomized controlled trials*: Well-designed randomized controlled trials (RCTs) are needed to generate high-quality evidence comparing community-based interventions – such as home-based chemotherapy, telehealth, and psychosocial support – with traditional hospital-centric care. Such trials will clarify the impact of these models on clinical outcomes, treatment adherence, quality of life, and patient satisfaction[73].
2. *Cost-effectiveness analyses*: Comprehensive economic evaluations are essential to determine the financial sustainability of community-based leukemia care programs. Understanding cost savings from reduced hospitalizations, decreased emergency visits, and improved treatment adherence will inform policymakers and healthcare systems on scalable implementation strategies[74].
3. *Implementation research*: Studies assessing the practical aspects of integrating community-based leukemia care into diverse healthcare settings are necessary. This includes identifying barriers and facilitators in different socio-economic and cultural contexts, evaluating workforce training needs, and optimizing care coordination between hospitals, community health workers, and telehealth systems[75].
4. *Equitable access and scalability*: Future research should ensure that community-based models are adaptable to resource-limited and underserved regions, including Africa and rural areas worldwide. Pilot programs should explore culturally tailored interventions and strategies to reduce disparities in care delivery[76].

## Recommendations

Based on the current evidence and emerging best practices in community-based leukemia management for older adults, we propose the following recommendations:

1. *Prioritize patient-centered community interventions*: Implement home-based chemotherapy, telehealth consultations, and community nursing programs as core strategies to reduce hospital dependency, enhance adherence, and improve quality of life for older adults.
2. *Strengthen training and workforce capacity*: Ensure that healthcare providers, including community nurses and health workers, are adequately trained in leukemia management, safe chemotherapy administration, telehealth protocols, and psychosocial support delivery.
3. *Integrate hospice and palliative care*: Early integration of palliative and hospice care into community-based programs should be standard, providing symptom management, psychosocial support, and guidance on advanced care planning, while complementing hospital-based treatments.
4. *Enhance access in resource-limited settings*: Tailor community-based interventions to local contexts, particularly in low-resource regions such as Africa, by leveraging existing community health structures, NGOs, and telemedicine platforms. Address barriers including infrastructure gaps, financial constraints, and workforce shortages.
5. *Promote multidisciplinary collaboration*: Foster collaboration between hospitals, community health teams, telehealth providers, and caregivers to ensure seamless, coordinated care and timely escalation of complications.
6. *Support evidence generation*: Encourage well-designed RCTs, cost-effectiveness studies, and implementation research to evaluate the scalability, feasibility, and long-term outcomes of community-based leukemia care models globally.
7. *Leverage technology and innovation*: Utilize telemedicine, remote monitoring devices, and digital health tools to extend care, track outcomes, and facilitate patient engagement, particularly for older adults with limited mobility or those in rural regions.
8. *Engage patients and caregivers*: Involve patients and caregivers in decision-making, care planning, and education to enhance adherence, satisfaction, and overall outcomes.

## Conclusion

Community-based approaches to leukemia management offer a promising pathway to optimize care for older adults, particularly in the context of global aging and the growing burden of comorbidities. By integrating home-based chemotherapy, telehealth interventions, psychosocial support, and hospice/palliative care, these models extend the reach of hospital-based services, enhance treatment adherence, reduce hospitalizations, and improve quality of life. Addressing challenges in resource-limited and underserved regions, while strengthening workforce capacity and infrastructure, is critical for equitable implementation. Future research – including randomized controlled trials, cost-effectiveness analyses, and implementation studies – will be essential to establish best practices, scalability, and long-term outcomes. Ultimately, community-centered leukemia care represents a patient-focused, sustainable approach capable of meeting the evolving needs of older adults worldwide.

## References

[1] Miranda-Filho A Piñeros M Ferlay J. Epidemiological patterns of leukaemia in 184 countries: a population-based study. Lancet Haematol 2018;5:14–24. 10.1016/S2352-3026(17)30232-6 29304322

[2] Zjablovskaja P Florian MC. Acute myeloid leukemia: aging and epigenetics. Cancers (Basel) 2019;12:103. 31906064 10.3390/cancers12010103 PMC7017261

[3] Bispo JA Pinheiro PS Kobetz EK. Epidemiology and etiology of leukemia and lymphoma. Cold Spring Harb Perspect Med 2020;10:a034819. 31727680 10.1101/cshperspect.a034819 PMC7263093

[4] Gri N Longhitano Y Zanza C. Acute Oncologic complications: clinical–therapeutic management in critical Care and Emergency Departments. Curr Oncol 2023;30:7315–34. 37623012 10.3390/curroncol30080531 PMC10453099

[5] Laribi K Sobh M Ghez D. Impact of age, functional status, and comorbidities on quality of life and outcomes in elderly patients with AML. Ann Hematol 2021;100:1359–76. 33796898 10.1007/s00277-020-04375-x

[6] Yamabe K Inoue S Hiroshima C. Epidemiology and burden of multiple myeloma in Japan: a systematic review. Value Health 2015;18:A449.

[7] Nasr IH Manson AL Al Wahshi HA. Optimizing hereditary angioedema management through tailored treatment approaches. Expert Rev Clin Immunol 2016;12:19–31. 26496459 10.1586/1744666X.2016.1100963

[8] Schattner A. The spectrum of hospitalization-associated harm in the elderly. Eur J Intern Med 2023;115:29–33. 37391309 10.1016/j.ejim.2023.05.025

[9] Paul CL Hall AE Carey ML. Access to care and impacts of cancer on daily life: do they differ for metropolitan versus regional hematological cancer survivors? J Rural Health 2013;29:43–50. 10.1111/jrh.12020 23944279

[10] Charlton M Schlichting J Chioreso C. Challenges of rural cancer care in the United States. Oncology. 2015;29:633–40. 26384798

[11] Balogun OD Mustapha AY Tomoh BO. Patient-centered care models: a review of their influence on healthcare management practices. J Front Multidiscip Res 2024;2:28–35.

[12] Kirsten S Laidsaar-Powell R Shaw JM. The holistic model of leukaemia survivorship care: derived from a qualitative exploration of leukaemia survivorship. Support Care Cancer 2025;33:1–2. 10.1007/s00520-025-09382-0 PMC11953203 40153078

[13] Liu JC Cheng CY Cheng TH. Unveiling the potential: remote monitoring and telemedicine in shaping the future of heart failure management. Life 2024;14:936. 39202678 10.3390/life14080936 PMC11355081

[14] Alhajri N Simsekler MC Alfalasi B. Exploring quality differences in telemedicine between hospital outpatient departments and community clinics: cross-sectional study. JMIR Med Inform 2022;10:e32373. 34978281 10.2196/32373 PMC8849258

[15] Dionne-Odom JN Currie ER Johnston EE. Supporting family caregivers of adult and pediatric persons with leukemia. Sem Oncol Nurs 2019;35:150–954. 10.1016/j.soncn.2019.150954 31753704

[16] Dhakal P Armitage JO, Bhatt VR. Academic and community cancer center collaboration in acute myeloid leukemia: a road map to optimize care delivery. Leuk Lymphoma. 2025;66:1389–99. 10.1080/10428194.2025.2484640 40168589

[17] Obeagu EI. World Health Organization (WHO)’s vision for a leukemia-free Africa: opportunities and challenges-a narrative review. Ann Med Surg 2025;87:10–97. 10.1097/MS9.0000000000003553 PMC12401219 40901113

[18] Bosserman LD Cianfrocca M Yuh B. Integrating academic and community cancer care and research through multidisciplinary oncology pathways for value-based care: a review and the city of hope experience. J Clin Med 2021;10:188. 33430334 10.3390/jcm10020188 PMC7825796

[19] Petersen CL Weeks WB Norin O. Development and implementation of a person-centered, technology-enhanced care model for managing chronic conditions: cohort study. JMIR Mhealth Uhealth 2019;7:e11082. 30892274 10.2196/11082 PMC6446154

[20] Kale S Hirani S Vardhan S. Addressing cancer disparities through community engagement: lessons and best practices. Cureus 2023;15:e43445. 10.7759/cureus.43445 PMC10498131 37711952

[21] Brailsford SC Lattimer VA Tarnaras P. Emergency and on-demand health care: modelling a large complex system. J Oper Res Soc 2004;55:34–42.

[22] Lee EK Chen CH Pietz F. Modeling and optimizing the public-health infrastructure for emergency response. Interfaces 2009;39:476–90.

[23] Krishnaswami J Sardana J Daxini A. Community-engaged lifestyle medicine as a framework for health equity: principles for lifestyle medicine in low-resource settings. Am J Lifestyle Med 2019;13:443–50. 31523209 10.1177/1559827619838469 PMC6732871

[24] Pratt R Gyllstrom B Gearin K. Identifying barriers to collaboration between primary care and public health: experiences at the local level. Public Health Rep 2018;133:311–17. 29614236 10.1177/0033354918764391 PMC5958390

[25] Hsu T Soto-perez-de-celis E Burhenn PS. Educating healthcare providers in geriatric oncology–a call to accelerate progress through identifying the gaps in knowledge. J Geriatr Oncol 2020;11:1023–27. 31732446 10.1016/j.jgo.2019.10.020 PMC9435653

[26] Ganesh A. Community-based health care models for the older persons. J Indian Acad Geriatr 2023;19:153.

[27] Jones CH Dolsten M. Healthcare on the brink: navigating the challenges of an aging society in the United States. Npj Aging 2024;10:22. 38582901 10.1038/s41514-024-00148-2 PMC10998868

[28] White C. The future awakens: a report on the 2016 Vizient clinical connections summit. Am J Med Qual 2017;32:3S–0S. 28467856 10.1177/1062860617701070

[29] Tulchinsky TH Varavikova EA. Organization of public health systems. New Public Health 2014;10:535.

[30] Robottom N. Oncology: establishing a community-based service. Cancer Nurs Pract 2013;12:14–20.

[31] Evans JM Qiu M MacKinnon M. A multi-method review of home-based chemotherapy. Eur J Cancer Care (Engl) 2016;25:883–902. 26545409 10.1111/ecc.12408

[32] Nørskov KH Overgaard D Lomborg K. Patients’ experiences and social support needs following the diagnosis and initial treatment of acute leukemia - A qualitative study. Eur J Oncol Nurs 2019;41:49–55. 31358257 10.1016/j.ejon.2019.05.005

[33] Hàshim SH Elsied MS Alqhtani RM. The impact of leukemia on child development: a holistic approach to care. Metall Mater Eng 2024;30:491–506.

[34] Bei E Morrison V Zarzycki M. Barriers, facilitators, and motives to provide distance care, and the consequences for distance caregivers: a mixed-methods systematic review. Soc Sci Med 2023;321:115782. 36801750 10.1016/j.socscimed.2023.115782

[35] Dossa A Bokhour B Hoenig H. Care transitions from the hospital to home for patients with mobility impairments: patient and family caregiver experiences. Rehabil Nurs J 2012;37:277–85. 10.1002/rnj.047 23212952

[36] Vaughn JE Buckley SA Walter RB. Outpatient care of patients with acute myeloid leukemia: benefits, barriers, and future considerations. Leuk Res 2016;45:53–58. 27101148 10.1016/j.leukres.2016.03.011 PMC5383350

[37] Blackburn LM Bender S, Brown S. Acute leukemia: diagnosis and treatment. Sem Oncol Nurs 2019;35:150950. 10.1016/j.soncn.2019.150950 31757585

[38] Gordon B Mason B Smith SL. Leveraging telehealth for delivery of palliative care to remote communities: a rapid review. J Palliat Care 2022;37:213–25. 33730904 10.1177/08258597211001184 PMC9286776

[39] Lee D. Strategies for technology-driven service encounters for patient experience satisfaction in hospitals. Technol Forecasting Social Change 2018;137:118–27.

[40] Molica S Allsup D. Chronic lymphocytic leukemia care and beyond: navigating the needs of long-term survivors. Cancers (Basel) 2025;17:119. 39796746 10.3390/cancers17010119 PMC11720366

[41] Cavusoglu H. Problems related to the diagnosis and treatment of adolescents with leukemia. Issues Compr Pediatr Nurs 2000;23:15–26. 11011660 10.1080/014608600265183

[42] Meier C Taubenheim S Lordick F. Depression and anxiety in older patients with hematological cancer (70+)–Geriatric, social, cancer-and treatment-related associations. J Geriatr Oncol 2020;11:828–35. 31831361 10.1016/j.jgo.2019.11.009

[43] Loh KP Abdallah M Kumar AJ. Health-related quality of life and treatment of older adults with acute myeloid leukemia: a Young International Society of Geriatric Oncology review paper. Curr Hematol Malig Rep 2019;14:523–35. 31776773 10.1007/s11899-019-00552-6 PMC6938300

[44] Pailler ME Johnson TM Zevon MA. Acceptability, feasibility, and efficacy of a supportive group intervention for caregivers of newly diagnosed leukemia patients. J Psychosoc Oncol 2015;33:163–77. 25587747 10.1080/07347332.2014.992086

[45] Yuan C Wang Z Xu X The lived experience of psychological resilience in parents of children with leukemia: a qualitative study.

[46] Keramatikerman M Varaei S Vaezi M. Peer support-based online education, burden of care and quality of life among family caregivers of patients with leukaemia: non-randomised clinical trial. BMJ Support Palliat Care 2024;14:e2827–35. 10.1136/spcare-2023-004610 PMC11671940 38272654

[47] Jonas DF Steineck A Johnson JA. Palliative care for special populations: pediatrics. Palliative Care Hematol Malig Ser Blood Disord 2023;28:255–71.

[48] Barnett M Breen KE Kennedy JA. Psychosocial interventions and needs among individuals and families with Li-Fraumeni syndrome: a scoping review. Clin Genet 2022;101:161–82. 34355387 10.1111/cge.14042

[49] San Juan AF Chamorro-Viña C Maté-Muñoz JL. Functional capacity of children with leukemia. Int J Sports Med 2008;29:163–67. 17879894 10.1055/s-2007-964908

[50] Randall J Gordon A Boyle C. Integrating social work throughout the hematopoietic cell transplantation trajectory to improve patient and caregiver outcomes. Transplant Cell Ther 2025;31:353.e1–353.e12. 10.1016/j.jtct.2025.03.013 PMC12146069 40120982

[51] Jepsen LØ Friis LS Hoybye MT. Rehabilitation during intensive treatment of acute leukaemia including allogenic stem cell transplantation: a qualitative study of patient experiences. BMJ Open 2019;9:e029470. 10.1136/bmjopen-2019-029470 PMC6886906 31727647

[52] Bryant AL Deal AM Battaglini CL. The effects of exercise on patient-reported outcomes and performance-based physical function in adults with acute leukemia undergoing induction therapy: exercise and quality of life in acute leukemia (EQUAL). Integr Cancer Ther 2018;17:263–70. 28627275 10.1177/1534735417699881 PMC6041904

[53] Mustian KM Sprod LK Janelsins M. Exercise recommendations for cancer-related fatigue, cognitive impairment, sleep problems, depression, pain, anxiety, and physical dysfunction: a review. Oncol Hematol Rev 2012;8:81. 23667857 10.17925/ohr.2012.08.2.81 PMC3647480

[54] Petruseviciene D Surmaitiene D Baltaduoniene D. Effect of community-based occupational therapy on health-related quality of life and engagement in meaningful activities of women with breast cancer. Occup Ther Int 2018;2018:6798697. 10.1155/2018/6798697 PMC5932445 29849515

[55] Santa Mina D Au D Brunet J. Effects of the community-based wellspring cancer exercise program on functional and psychosocial outcomes in cancer survivors. Curr Oncol 2017;24:284. 29089795 10.3747/co.23.3585 PMC5659149

[56] Lynch MP DeDonato DM Kutney-Lee A. Geriatric oncology program development and gero-oncology nursing. Semin Oncol Nurs 2016;32:44–54. 26830267 10.1016/j.soncn.2015.11.006

[57] Chung YC. Education for homecare patients with leukemia following a cycle of chemotherapy: an exploratory pilot study. Oncol Nurs Forum 2008;35:E83–9.

[58] Wedding U. Palliative care of patients with haematological malignancies: strategies to overcome difficulties via integrated care. Lancet Health Longev 2021;2:746–53. 10.1016/S2666-7568(21)00213-0 36098031

[59] Brewster AL Yuan CT Tan AX. Collaboration in health care and social service networks for older adults: association with health care utilization measures. Med Care 2019;57:327–33. 30908380 10.1097/MLR.0000000000001097

[60] Mikhael J Darlington D Howell B. The benefits of telehealth in promoting equity in blood cancer care–results of a multi-stakeholder forum and systematic literature review. J Med Econ 2025;28:788–802. 40340653 10.1080/13696998.2024.2438561

[61] Lindeman DA Kim KK Gladstone C. Technology and caregiving: emerging interventions and directions for research. Gerontologist 2020;60:9–41. 10.1093/geront/gnz178 PMC7019659 32057082

[62] McCarthy PM Lamm RD Sade RM. Medical ethics collides with public policy: LVAD for a patient with leukemia. Ann Thorac Surg 2005;80:793–98. 16122431 10.1016/j.athoracsur.2005.05.081

[63] Waldron EA Janke EA Bechtel CF. A systematic review of psychosocial interventions to improve cancer caregiver quality of life. Psycho-Oncology 2013;22:1200–07. 22729992 10.1002/pon.3118

[64] Cronin RM Mayo-Gamble TL Stimpson SJ. Adapting medical guidelines to be patient-centered using a patient-driven process for individuals with sickle cell disease and their caregivers. BMC Hematol 2018;18:12. 29977566 10.1186/s12878-018-0106-3 PMC5994026

[65] Mikhael JR Sullivan SL Carter JD. Multisite quality improvement initiative to identify and address racial disparities and deficiencies in delivering equitable, patient-centered care for multiple myeloma – Exploring the differences between academic and community oncology centers. Curr Oncol 2023;30:1598–613. 36826084 10.3390/curroncol30020123 PMC9955622

[66] Nakamura S Maeda Y Hori T. Hematology in community medical care. J Med Invest 2025;72:21–25. 40268449 10.2152/jmi.72.21

[67] Jillella AP Cortes JE Kota VK. Optimizing management of acute leukemia in community centers and when to refer Hematology 2014. Am Soc Hematol Educ Program Book 2020;2020:123–28. 10.1182/hematology.2020000096 PMC7727530 33275676

[68] Mansukhani RP Bridgeman MB Candelario D. Exploring transitional care: evidence-based strategies for improving provider communication and reducing readmissions. Pharm Ther 2015;40:690. PMC4606859 26535025

[69] Alkema GE Reyes JY Wilber KH. Characteristics associated with home-and community-based service utilization for Medicare managed care consumers. Gerontologist 2006;46:173–82. 16581881 10.1093/geront/46.2.173

[70] Mah JC Stevens SJ Keefe JM. Social factors influencing utilization of home care in community-dwelling older adults: a scoping review. BMC Geriatr 2021;21:145. 33639856 10.1186/s12877-021-02069-1 PMC7912889

[71] Afriyie DO Damoah KA Mussa EC. Barriers and facilitators to health services utilization among households with free community-based health insurance enrolment in Ethiopia: a qualitative study. SSM-Health Syst 2025;4:1.

[72] Goodridge D Marciniuk D. Rural and remote care: overcoming the challenges of distance. Chron Respir Dis 2016;13:192–203. 26902542 10.1177/1479972316633414 PMC5734598

[73] Tang X Chen SQ Huang JH. Assessing the current situation and the influencing factors affecting perceived stigma among older patients after leukemia diagnosis. World J Psychiatry 2024;14:812. 38984333 10.5498/wjp.v14.i6.812 PMC11230094

[74] Everall AC Guilcher SJ Cadel L. Patient and caregiver experience with delayed discharge from a hospital setting: a scoping review. Health Expect 2019;22:863–73. 31099969 10.1111/hex.12916 PMC6803563

[75] Bertucci F Le Corroller-Soriano AG Monneur-Miramon A. Outpatient cancer care delivery in the context of e-oncology: a french perspective on “cancer outside the hospital walls.” Cancers (Basel) 2019;11:219. 30769858 10.3390/cancers11020219 PMC6406853

[76] Agha RA Mathew G Rashid R. TITAN Group. Transparency in the Reporting of Artificial Intelligence – the TITAN Guideline. Prem J Sci 2025;10:100082.

